# Multifactorial assessment of braking time predictors in a driving simulator among older adults according to gender

**DOI:** 10.1016/j.clinsp.2024.100405

**Published:** 2024-07-04

**Authors:** Maria Eugenia Mayr De Biase, Angelica Castilho Alonso, Reinaldo Nonato da Silva, Sara Moutinho Soares, Alexandra Carolina Canonica, Alessandra Pricila dos Reis Belini, Jose Maria Soares-Junior, Edmund Chada Baracat, Alexandre Leopold Busse, Wilson Jacob-Filho, Guilherme Carlos Brech, Júlia Maria D'Andrea Greve

**Affiliations:** a Laboratory Study of Movement, Instituto de Ortopedia e Traumatologia do Hospital das Clínicas (IOT-HC) da Faculdade de Medicina da Universidade de São Paulo (FMUSP), São Paulo, SP, Brazil; b Graduate Program in Aging Sciences, Universidade São Judas Tadeu (USJT), São Paulo, SP, Brazil; c Disciplina de Ginecologia, Departamento de Obstetrícia e Ginecologia, Hospital das Clínicas, Faculdade de Medicina da Universidade de São Paulo (FMUSP), São Paulo, SP, Brazil; d Departamento de Geriatria da Faculdade de Medicina da Universidade de São Paulo (FMUSP), São Paulo, SP, Brazil

**Keywords:** Reaction Time, Older Adults, Driving a Vehicle, Aging

## Abstract

**Context:**

Vehicle driving depends on the integration of motor, visual, and cognitive skills to respond appropriately to different situations that occur in traffic.

**Objectives:**

To analyze a model of performance predictor for braking time in the driving simulator, using a battery of tests divided by gender. Methods: Selected were 100 male drivers with a mean age of 72.6 ± 5.7 years. Sociodemographic variables, braking time in the driving simulator, and motor, visual, and cognitive skills were evaluated.

**Results:**

Comparing genders, men were older than women (*p* = 0.002) and had longer driving times (*p =* 0.001). Men had more strength in hand grip (*p* ≤ 0.001). In the linear regression analysis, the model explained 68 % of the braking time in men and 50.8 % in women. In the stepwise multiple linear regression analysis, the variable that remained in the model was the strength of the right plantar flexors, which explained 13 % of the braking time in women and men, and the cognitive variables explained 38.9 %.

**Conclusion:**

Sociodemographic, motor, visual, and cognitive variables, explained a substantial portion of the variability in braking time for both older women and men, the specific variables driving this performance differed between the sexes. For older women, factors such as muscle strength emerged as critical determinants of braking ability, highlighting the importance of physical health in maintaining driving skills. On the other hand, cognitive conditions emerged as the primary predictor of braking performance in older men, underscoring the role of mental acuity and decision-making processes in safe driving.

## Highlights

• Sociodemographic, motor, visual and cognitive variables can predict the braking time.
• In women muscle strength for predict the braking time.
• In men cognitive conditions predict the braking time.

## Introduction

Longevity is humanity's greatest achievement; however, it generates challenges and concerns that affect society. The increase in population leads to an increase in older adult drivers. In Brazil, drivers over 65 years of age went from four and a half million to eight million drivers between 2015 and 2019 (National Association of DETRANS). In 2020, there were almost 48 million licensed drivers ages 65 and older in the United States. This is a 68 % increase since 2000.^1^

Vehicular driving of older adults preserves autonomy and social interaction, maintaining involvement with the environments that allow an active aging process.^2^ Not driving is associated with the perception of losing control over one's life, the appearance of depressive symptoms, and isolation.^2^^,^^3^

Driving is a complex activity requiring rapid and continuous integration of cognitive, visual, and motor skills. Aging can compromise the safety of vehicular driving by increasing the braking time.^4^ The braking time of older adult drivers is 17 % longer than that of young people. Zahabi et al.,^5^ evaluated attention and neural efficiency in vehicular direction and showed that learning and performance differ according to gender. Women require less mental effort in driving, and men use more visual attention and a faster activation of car features.

Many factors can influence braking performance in older drivers since older adults face specific challenges related to traffic safety. Additionally, considering the biological, behavioral, and socioeconomic e sociodemographic differences between men and women in older adults, it is important to investigate how these factors may affect braking time, thereby contributing to the development of more effective road safety interventions and policies looking forward to the specific needs of each group.

The present study aimed to analyze a multifactorial model for evaluating braking time in the driving simulator of older adult drivers and to verify the difference between men and women.

## Methods

### Experimental design, local and ethics

This cross-sectional study was conducted at the Motion Study Laboratory of the Institute of Orthopedics and Traumatology, Hospital das Clínicas, University of São Paulo School of Medicine, approved by the Ethics Research Committee CAAE: 42276214.9.0000.0065

### Participants

One hundred drivers of both genders were divided into two groups: 1) Women's group: 50 women aged 70.8 ± 5.7 years and 2) Men's group: 50 men aged 74.3±5.5 years. The inclusion criteria were: being over 60; having a valid driver's license and driving at least two days a week at the time of the assessment; not presenting limitations of the ankle, knee, and hip joint movement; normal gait; without previous injuries or surgery on the spine, lower and upper limbs; Mini Mental Health State Exam test (literate ≥ 24 points)^13^; do not use drugs that may alter the ability to drive.

### Procedures

All subjects agreed to participate in the study by reading and signing the informed consent form. Afterward, they answered a questionnaire with personal information, sociodemographics, and driving history.

## Assessments

### Muscular strength

The isokinetic dynamometer evaluated strength in the plantar flexor muscle (Biodex System 2, USA). The volunteers were seated with support in the distal region of the thigh, the sole supported on a rigid plate, and the knee was maintained with 30° of flexion. The subject remained in position by two chest belts, a pelvic belt, and velcro straps over the distal thigh and metatarsal area in the dorsal region of the foot. The authors requested three submaximal attempts to familiarize them with the equipment. Two tests were performed with five repetitions each, at an angular speed of 30°/s, always starting with the dominant limb. Verbal stimuli were given throughout the trials to motivate participants. The data analysis was considered the measure of the second test.^6^

Palmar Grip Strength ‒ measured on the Jamar® dynamometer on the dominant and non-dominant limbs. The individual remained seated with the arms parallel to the body, adducted shoulder, with 90º flexion of the elbow, forearm, and wrist in a neutral position. Three measures were interspersed between the dominant and non-dominant hand, with a one-minute interval between each attempt and the average obtained in Kilogram-Force (Kg/f).^7^

### Function tests

Mobility and balance ‒ “Time up and Go” Test (TUG), which measures the time (seconds) for the individual to get up from a chair, walk three meters, turn around, return to the chair, and sit down again at the usual speed. Additionally, men performed the TUGT with a double task (“cognitive” TUGT), which associates motor activities with verbalizing words: colors in the first test, animals in the second, and fruits in the third. The average time of the three trials was computed.^8^

Functional Reach Test ‒ the Functional Reach Test ‒ assesses the ability of the trunk to move forward within the limits of stability. The individuals lean on starting from the orthostatic position, perpendicular to the wall, with 90° flexion of the shoulder, elbows extended, and heels together. The variable used is the distance covered by the third metacarpal along the horizontal axis measured with a tape measure. Three attempts were made, and the average was calculated.^9^

Articular amplitude: 1) Rotation of the cervical spine (0°‒55°) ‒ Individual sitting with the head and neck in an anatomical position. The side to be evaluated is rotated. The goniometer's fixed arm is positioned in the sagittal suture (center of the head), and the mobile arm must be placed in the sagittal suture at the end of the movement. Shoulder Flexion – (0‒180°). The volunteer is seated, with the arms by the body and elbows extended. The fixed arm of the goniometer is placed along the middle axillary line of the trunk, pointing to the greater trochanter of the femur and the mobile component of the goniometer on the lateral surface of the humeral body facing the lateral epicondyle of the wrist.^10^

### Visual test

The Raizamed 2000 equipment was used. Visual acuity: It was measured using the Snellen optometric scale; Measured using the Snellen optometric scale, which consists of a set of letters that become progressively smaller from top to bottom. On this scale, normal visual acuity is called 20/20. The score falls according to the last line that the volunteer sees correctly.

Visual campimetry ‒ measurement of both eyes' unilateral 90° and 180° temporal field of vision.

### Cognitive assessments

Trail Making Test (Trail Making B Test) Part B assesses alternate attention, consists of linking numbers and letters in an orderly and consecutive way, and evaluates the inhibitory control of responses.^11^

Montreal Cognitive Assessment” (MoCA) ‒ an instrument developed to screen for mild cognitive impairment and access different cognitive domains: attention and concentration, executive functions, memory, language, visual-constructive skills, conceptualization, calculation, and guidance. The total score is 30 points. A score of 26 or more is considered normal.^12^

Mini-Mental State Examination (MMSE) ‒ Test with 11 items that can reach 30 points and assess cognitive function. They address issues related to recent memory and the recording of immediate memory, temporal and spatial orientation, attention, calculation, and language ‒ aphasia, apraxia, and constructional skills.^13^

### Driver questionnaire

Self-perception of disability based on the “Can Drive” questionnaire^14^ 30 questions were created in the following domains: behavior: how to behave in the face of various stimuli; perception: the way of conceptualizing, judging or qualifying something; cognition: the process of knowing or acquiring knowledge; vision: perception through the eyes; motor: body movements and asked if he had difficulty or not, it was calculated with a point for each statement ‒ *I have difficulty*.

### Driving simulator test

Time to brake the car: The driving simulator “Car-Simulator Trainer ‒ Type F12PT” (FoerstGMBh) was used. The route was visualized on three LCD TV monitors (42′). On the road, at random, a sign labeled “STOP” appears, and the volunteer must brake and stop the vehicle completely. The braking time is measured from the moment the volunteer applies the brake automatically by the equipment. The command was repeated five times. The arithmetic mean of the five tests was used.^8^

### Statistical analysis

Descriptive variables were described by mean and standard deviation, divided by gender. The Komogorov-Smirnov test was used to verify the normality of the data. Multiple linear regression (forward mode) was performed to investigate whether the independent variables predict the braking time, separated by sex. The variables were included in the following order: Model 1 ‒ Sociodemographic, Model 2 ‒ Sociodemographic, and motor. Model 3 ‒ Sociodemographic, motor, and visual; and Model 4 ‒ Sociodemographic, motor, visual and cognitive. The stepwise multiple linear regression model was used to analyze the performance predictor variables associated with the domains. For this, all variables that showed *p* ≤ 0.05 in the correlation coefficient analysis were chosen. Next, they were ordered from the lowest *p*-value to the highest. The multiple modeling process was the “stepwise forward selection”. Those with a value of *p* ≤ 0.05 remained in the model. The data were analyzed using the SPSS 20.0 program.

## Results

Men were older than women (*p =* 0.002) and had longer driving times (*p =* 0.001). In the motor domain, men were stronger than women (*p* ≤ 0.001) (Table 1).

**Table 1:** Comparison between genders of demographic data and domains: motor, visual, cognitive.

	Women (*n* = 50) M (sd)	Men (*n* = 50) M (sd)	*p*-value
**Demographic data**			
Age (years)	70.8 (5.5)	74.3 (5.5)	0.002*
Schooling (years)	12.1 (2.9)	12.5 (2.8)	0.444
Driving time (years)	45.9 (5.8)	50.5 (7.3)	0.001*
**Motor Domain**			
DS handgrip test (kg/f)	25.7 (4.7)	38.2 (8.6)	*p* ≤ 0.001*
NDS handgrip test (kg/f)	23.8 (5.4)	34.2 (8.0)	*p* ≤ 0.001*
Functional Reach Test (cm)	31.4 (6.4)	33.0 (5.4)	0.195
DP Peak torque corrected body weight (%)	74.3 (27.3)	81.9 (28.5)	0.180
Total work DP 5 repetitions (J)	24.9 (0.0)	24.9 (0.3)	0.429
Time Up and Go (s)	8.7 (1.3)	8.6 (2.2)	0.928
R Shoulder flexion (°)	165.0 (17.9)	160.2 (18.0)	0.187
R Cervical rotation (°)	69.7 (10.4)	67.8 (12.4)	0.419
L Shoulder flexion (°)	165.0 (17.2)	158.7 (18.7)	0.083
L Cervical rotation (°)	72.9 (18.7)	69.4 (12.5)	0.269
**Visual Domain**			
Snelling LS	4.1 (2.9)	3.6 (3.1)	0.419
Snelling DR	4.0 (3.1)	4.2 (3.1)	0.814
Snelling Binocular	1.1 (1.3)	1.1 (1.3)	0.940
Right eye Campimetry (°)	85.9 (7.5)	85.8 (7.7)	0.948
Left eye Campimetry (°)	85.8 (6.7)	86.8 (5.0)	0.402
**Cognitive Domain**			
MoCA	23.8 (3.3)	22.8 (3.6)	0.141
Trail make B – errors	5.1 (6.4)	6.0 (6.5)	0.489
Trail make B- time (s)	136.9 (65.1)	171.6 (129.9)	0.095
Cognitive Time Up and Go (s)	10.8 (2.7)	10.8 (2.7)	0.539
Mini-Mental State Exam	27.9 (1.7)	28.0 (1.8)	0.956
Self-perception of difficulty	5.0 (3.0)	4.7 (3.0)	0.601
**Braking time (s)**	0.97 (0.1)	0.92 (0.1)	0.131

DS, Dominant Side; NDS, Non-Dominant Side; RP, Right Plantar flexors; R, Right; L, Left; LE, Left Eye; RE, Right Eye; MoCA, Montreal Cognitive Assessment.

In the analysis of linear regression for WOMEN having as a dependent variable braking time, Model 1, the sociodemographic variables: age, education, and driving time explain 7.3 % of the braking time. In Model 2, sociodemographic variables and motor domains involving muscle strength, balance, and flexibility explain 31.2 % of the braking time. In Model 3, the independent sociodemographic variables and motor and visual domains explain 40.3 % of the braking time. Finally, in Model 4, the independent sociodemographic variables, motor, visual and cognitive domains explained 50.8 % of the braking time (Table 2).

**Table 2:** Linear regression analysis for braking time with the sociodemographic, motor, visual and cognitive status domains of women (*n* = 50).

Model for braking time versus women								
Variables	Model 1		Model 2		Model 3		Model 4	
Braking time	β	EP	β	EP	β	EP	β	EP
Demographic data								
Age (years)	0.010	0.006	0.011	0.007	0.002	0.009	0.004	0.010
Schooling (years)	−0.005	0.008	−0.004	0.009	−0.007	0.010	−0.002	0.012
Driving time (years)	−0.006	0.006	−0.004	0.007	0.000	0.008	0.002	0.008
**Motor Domain**								
DS handgrip (kg/f)			0.006	0.010	0.004	0.011	−0.010	0.012
NDS handgrip (kg/f)			−0.015	0.009	−0.011	−0.009	−0.011	0.011
Functional reach (cm)			0.004	0.006	0.005	0.006	0.005	0.007
PT/BW PFD (%)			−0.002	0.001	−0.002	0.001	−0.002	0.002
TT PFD (J)			0.106	0.099	0.053	0.110	−0.069	0.130
TUG (s)			−0.015	0.024	−0.010	0.027	0.003	0.031
R Shoulder flexion (°)			−0.002	0.003	−0.002	0.003	0.001	0.004
R Rotation (°)			−0.001	0.003	−0.001	0.004	−0.004	0.004
L Shoulder flexion (°)			0.004	0.003	0.004	0.003	0.002	0.004
L Rotation (°)			−0.001	0.003	0.000	0.002	−0.001	0.002
**Visual Domain**								
Snelling LE					0.054	0.068	0.060	0.077
Snelling RE					0.067	0.066	0.068	0.077
Snelling binocular					0.004	0.070	0.002	0.081
RE Campimetry (°)					0.002	0.006	0.002	0.007
LE Campimetry (°)					−0.006	0.007	−0.004	0.008
**Cognitive Domain**								
MoCA							−0.020	0.013
Trail make – errors							−0.008	0.008
Trail make ‒ time (s)							0.000	0.001
Cognitive TUG							0.008	0.019
Mini-Mental							−0.007	0.015
Self-perceived difficulties							0.004	0.012
***r* square**	**0.073**		**0.312**		**0.403**		**0.508**	

DS, Dominant Side; NDS, Non-Dominant Side; PT/BW, Peak Torque divided by Body Weight; RPF, Right Plantar Flexors; TW, Total Work; TUG, Time Up Go; R, Right; L, Left; LE, Left Eye; RE, Right Eye; MoCA, Montreal Cognitive Assessment.

In the analysis of linear regression for MEN with the dependent variable, braking time, Model 1, the sociodemographic variables: explained 12.0 % of the braking time. In Model 2, sociodemographic variables and motor domains explained 38.9 %. In Model 3, sociodemographic variables and motor and visual domains explained 55.0 %. Finally, in Model 4, the sociodemographic variables, motor, visual and cognitive domains explained 68.0 % of the braking time (Table 3).

**Table 3:** Linear regression analysis for braking time with sociodemographic domains; motor, visual and cognitive effects of men (*n* = 50).

Model for braking time versus men.								
Variables	Model 1		Model 2		Model 3		Model 4	
Braking time	β	EP	β	EP	β	EP	Β	EP
Demographic data								
Age (years)	0.011	0.005	0.010	0.006	0.004	0.007	0.005	0.008
Schooling (years)	0.000	0.008	0.010	0.009	0.005	0.009	0.006	0.011
Driving time (years)	−0.006	0.003	−0.008	0.004	−0.009	0.004	−0.007	0.005
**Motor Domain**								
DS handgrip (kg/f)			−0.003	0.006	0.000	0.006	−0.002	0.006
NDS handgrip (kg/f)			0.002	0.007	0.000	0.007	0.000	0.007
Functional reach (cm)			0.004	0.004	0.001	0.004	0.003	0.004
PT/BW PFD (%)			0.000	0.001	0.001	0.001	0.000	0.001
TT PFD (J)			−0.043	0.066	−0.092	0.074	−0.055	0.083
TUG (s)			0.010	0.012	0.017	0.013	−0.011	0.020
R Shoulder flexion (°)			−0.004	0.002	0.001	0.002	−0.001	0.003
R Rotation (°)			−0.001	0.003	0.003	0.004	−0.002	0.004
L Shoulder flexion (°)			−0.004	0.002	−0.003	0.002	−0.001	0.002
L Rotation (°)			−0.003	0.003	−0.005	0.003	−0.006	0.003
**Visual Domain**								
Snelling LE					−0.020	0.042	−0.021	0.044
Snelling RE					−0.029	0.045	−0.023	0.052
Snelling binocular					−0.019	0.053	−0.005	0.063
RE Campimetry (°)					0.002	0.003	0.000	0.004
LE Campimetry (°)					−0.018	0.007	−0.014	0.008
**Cognitive Domain**								
MoCA							−0.009	0.009
Trail make – errors							−0.004	0.004
Trail make ‒ time (s)							0.000	0.000
Cognitive TUG							0.026	0.021
Mini-Mental							0.019	0.015
Self-perceived difficulties							0.002	0.008
***r* square**	**0.120**		0.389		**0.550**		**0.680**	

DS, Dominant Side; NDS, Non-Dominant Side; PT/BW, Peak Torque divided by Body Weight; RPF, Right Plantar Flexors; TW, Total Work; TUG, Time Up Go; R, Right; L, Left; LE, Left Eye; RE, Right Eye; MoCA, Montreal Cognitive Assessment.

**Fig. 1:**
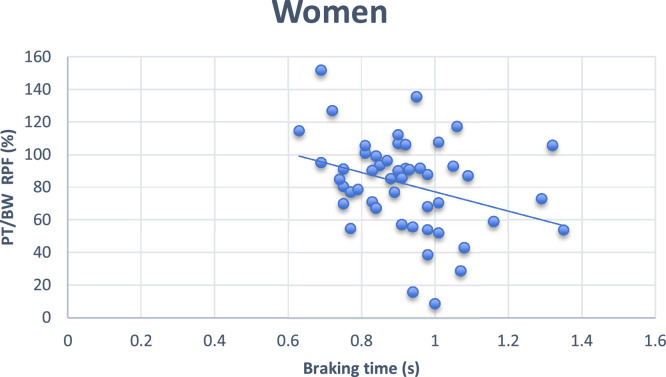
Scatter plot between braking time and women's PT/BW PFD. PT/BW, Peak Torque divided by Body Weight; RPF, Right Plantar Flexors; TW, Total Work; TUG, Time up Go; R, Right; L, Left; LE, Left Eye; RE, Right Eye; MoCA, Montreal Cognitive Assessment.

**Fig. 2:**
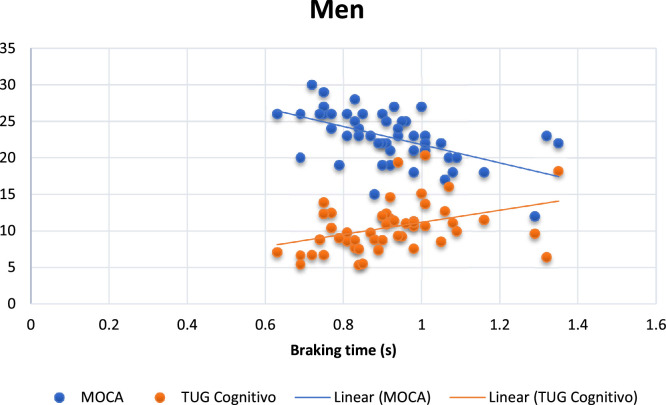
Scatter plot between braking time MOCA (blue) and Cognitive TUG (red) for men. TUG, Time up Go; MOCA, Montreal Cognitive Assessment. (For interpretation of the references to color in this figure legend, the reader is referred to the web version of this article.)

In the Multiple Stepwise linear regression analysis, the variable that remained in the model was the muscle strength of the right plantar flexors (PT/BW PFD %), predicting 13 % of the time to brake the car in women. In men, the variables in the cognitive MoCA and TUG remained in the model, explaining 38.9 % of the time braking the car (Table 4; Figs. 1 and 2).

**Table 4:** Multiple Stepwise Linear Regression for predictors of braking time in all men and divided by gender.

		β	Error Standard	*p*-value	*r* square
Women					
1	PT/BW PFD (%)	−0.002	0.001	0.01	0.130
**Men**					
1	MoCA	−0.023	0.005	0.000	0.289
2	MoCA	−0.021	0.005	0.000	0.389
	Cognitive TUG	0.015	0.005	0.009

## Discussion

The sociodemographic, motor, visual and cognitive variables were able to predict the performance in the braking time of drivers during the use of the driving simulator, showing the multifactorial and gender influence on the vehicle driving ability. The proposed model explained 68 % of the braking time for men and 50.8 % for women. The influence of the domains is different for the genders. Women are more affected by motor losses, particularly by decreased muscle strength, and men by cognitive losses in relation to braking time. This data allows for some inferences regarding the safety of men's driving vehicles: greater attention to women's physical and men's mental assessment.

When comparing genders, men were older, with longer driving times than women. In the regression analysis, these factors, plus schooling, were able to explain 7.3 % of the braking time in women and 12 % in men. Alonso et al.,^8^ affirm that the ability to drive declines with age but that this in itself does not determine competence in driving since aging is a heterogeneous process and affects individuals differently. Zhang et al.,^15^ showed that the reaction time to start braking and the act of braking with the driving simulator is related to increasing age (especially after 60 years) and the female gender. Men, in general, have been driving longer than women, a possible cultural fact related to the age of the sample where men were more encouraged to drive than women. Wayne et al.,^16^ refer that older men feel more comfortable and are more skilled in driving than women.

When overlapping sociodemographic data with motor domain variables, the model explains 31.2 of the braking time in women and 38.9 % in men. In the stepwise regression analysis, the plantar flexor muscle strength variable was the one that remained in the model, explaining 13 % of the braking time in women. Some characteristics specific to gender may be the origin of these findings.^8^ Women are more affected by sarcopenia than men: hormonal and dietary factors, which are seen in menopause, in addition to, of course, the lower physical constitution of women.^17^ Motor losses, more evident in women, can contribute to a greater perception of difficulties in driving. Muscle strength training could improve women's braking performance and increase vehicle driving safety.^8^

Lacherez et al.^18^ state that musculoskeletal functions impact driving ability, especially head and neck flexibility, which are associated with vehicular collisions. The reduction in muscle strength of the knee extensors and plantar flexors decreased motor coordination, and aging balance is associated with increased braking time in older adults.

The model's visual domain increase explained the braking time of 40.3 % in women and 55 % in men. Visual impairment can be corrected with lenses, but some serious losses can prevent vehicle driving. A study with simulators^19^ showed that visual and cognitive impairment is strongly associated with increased accidents among older adult drivers. Li et al.,^20^ in a study with simulators, showed that visual and cognitive impairment is associated with increased accidents among older adult drivers. Merickel et al.,^21^ refer that the loss of visual acuity impairs the perception of road signs or dangers, with sudden vehicle control maneuvers, increasing braking time and collision risks.

With the addition of the cognitive domain, the model explained the braking time of 50.8 % in women and 68 % in men. Wagner et al.^22^ define an executive function as the capacity to respond to new situations adaptively, including volition, planning, anticipation, and effective performance. Cognitive losses are very important for the safety of vehicular driving since they involve making decisions and actions that can prevent or cause accidents. In the current study, cognitive losses (cognitive TUG and MoCA), when analyzed in stepwise multiple linear regression, explained 38.9 % of the braking time in men, showing the importance of the ability to drive vehicles.

Men are stronger, and motor loss is more gradual. Still, they have more difficulty in the simultaneous execution of multiple tasks, hence probably the greatest effect of the cognitive decline in braking time, corroborating with Zahabi et al.,^5^ where women exhibited greater neural efficiency in vehicular driving in a driving simulator compared to men, since they are more trained in multiple tasks, common in their daily lives.^23^ Conversely, men exhibited greater activation of the right prefrontal cortex (visual attention and spatial memory), frequently using the vehicle's resources automatically.

The present study shows different behaviors in men and women about vehicular direction, similar to that of Canônica^24^^,^^25^ which showed that in women, the TUGT with the dual task (cognitive and motor), age, and muscular strength were the most important factors. For determinants of braking time in men, there was only the TUGT with a dual task. Men are stronger, and motor loss is more gradual, but they have more difficulty performing multiple tasks simultaneously, which is probably the greatest effect of cognitive decline. Also, is known that older men were stronger than older women during concentric isokinetics assessments at the ankle joint.^26^

Furthermore, Lo et al.^27^ highlighted the significance of driving frequency in relation to the connection between executive functions and both driving performance and behavior. Tasks requiring executive control are more indicative of driving capabilities in novice drivers, whereas the temperament of the driver, such as impulsiveness, is a stronger predictor of driving abilities in seasoned drivers. For this reason, different vehicle collision scenes can be related to driver's different active emergency responses.^28^

The study's limitations are related to the driving simulator, which, although a useful tool for studying driving performance, may not fully replicate real-life conditions. Participants' reactions may differ in a virtual environment compared to actual driving, which could impact the results. Despite including a variety of sociodemographic, motor, visual, and cognitive variables, there may be other important variables not considered in the study that could influence braking time for older drivers. Lastly, external factors such as environmental or traffic conditions may influence participants' driving performance in ways not accounted for in the study.

The reduction in the risk of infractions and collisions brings greater autonomy and independence to the older adults, with a positive impact on their quality of life and these gender-specific differences underscore the need for personalized interventions aimed at preserving safe driving performance as individuals age. For older women, interventions focusing on enhancing muscle strength and physical fitness could be particularly beneficial, potentially improving their ability to execute timely braking maneuvers. Meanwhile, interventions for older men may prioritize cognitive training and strategies to support mental agility and attention during driving.

## Conclusion

The results revealed that, although the proposed model, including sociodemographic, motor, visual, and cognitive variables, explained a substantial portion of the variability in braking time for both older women and men, the specific variables driving this performance differed between the sexes. For older women, factors such as muscle strength emerged as critical determinants of braking ability, highlighting the importance of physical health in maintaining driving skills. On the other hand, cognitive conditions emerged as the primary predictor of braking performance in older men, underscoring the role of mental acuity and decision-making processes in safe driving.

These gender-specific differences underscore the need for personalized interventions aimed at preserving safe driving performance as individuals age. For older women, interventions focusing on enhancing muscle strength and physical fitness could be particularly beneficial, potentially improving their ability to execute timely braking maneuvers. Meanwhile, interventions for older men may prioritize cognitive training and strategies to support mental agility and attention during driving.

## Authors’ contributions

Maria Eugenia Mayr De Biase: Writing-original draft; Investigation.

Angelica Castilho Alonso: Formal analysis.

Reinaldo Nonato da Silva: Writing-original draft.

Sara Moutinho Soares: Writing-original draft.

Alexandra Carolina Canonica: Writing-original draft; Investigation.

Alessandra Pricila dos Reis Belini: Writing-original draft.

Jose Maria Soares-Junior: Validation.

Edmund Chada Baracat: Validation.

Alexandre Leopold Busse: Methodology.

Wilson Jacob-Filho: Methodology.

Guilherme Carlos Brech: Writing-review & editing.

Júlia Maria D'Andrea Greve: Supervisor and Writing-review & editing.

## Funding

This study was financed by the Fundação de Amparo à Pesquisa (Foundation for Research Support) process nº 2012/20627-5 and Scientific Cooperation Agreement between Fundação de Amparo à Pesquisa do Estado de São Paulo (FAPESP) ‒ Brazil and University of Michigan ‒ EUA, process nº 13/50138-9.

## Declaration of competing interest

The authors declare no conflicts of interest.
